# Supplementary material for: Automated analysis of scanning electron microscopic images for assessment of hair surface damage
Source: R Soc Open Sci. 2020 Jan 15;7(1):191438. doi: 10.1098/rsos.191438 (PMC7029898; doi:10.1098/rsos.191438)
Supplement: Supplementary Figures S1 & S2 and Appendices 1 & 2 [file rsos191438supp1.pdf]

Automated analysis of scanning electron microscopic images for assessment of hair surface damage

Fanny Chu[1,2]*, Deon S. Anex[1], A. Daniel Jones[3], Bradley R. Hart[1]

[1]Lawrence Livermore National Laboratory, 7000 East Ave. Livermore, CA 94550, USA
[2]Department of Chemistry, Michigan State University, 578 S Shaw Ln East Lansing, MI 48824, USA
[3]Department of Biochemistry and Molecular Biology, 603 Wilson Road, East Lansing, MI 48824, USA

***Corresponding Author**
Fanny Chu
chu28@llnl.gov

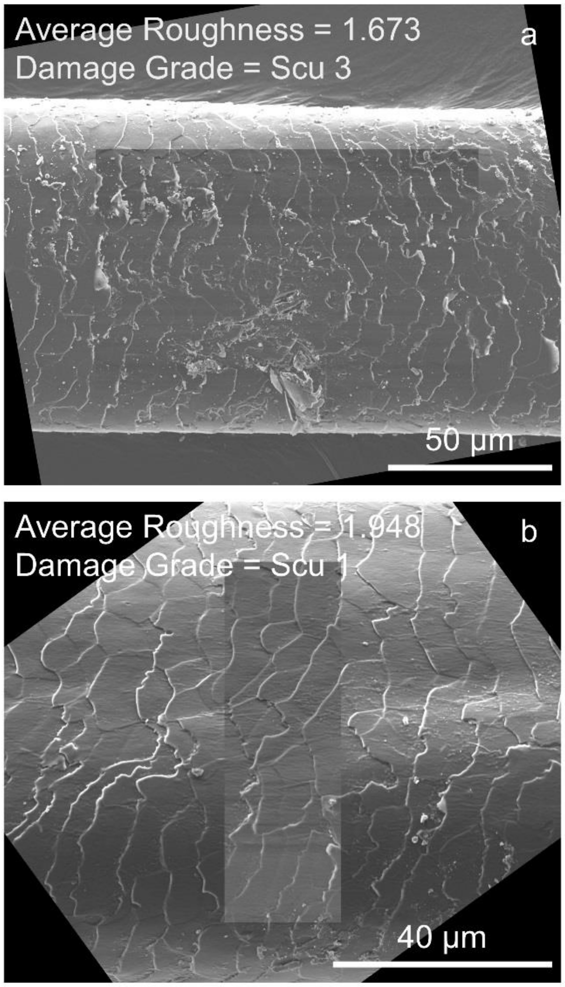

Figure S1. Example rotated SEM images with overlays of normalized regions of interest from (a) Hair Sample 1 and (b) Hair Sample 4 that exhibit similar average image roughness but vastly different extents of damage, as assessed with the SEM damage grade system. Average image roughness fails to effectively characterize hair surface damage.

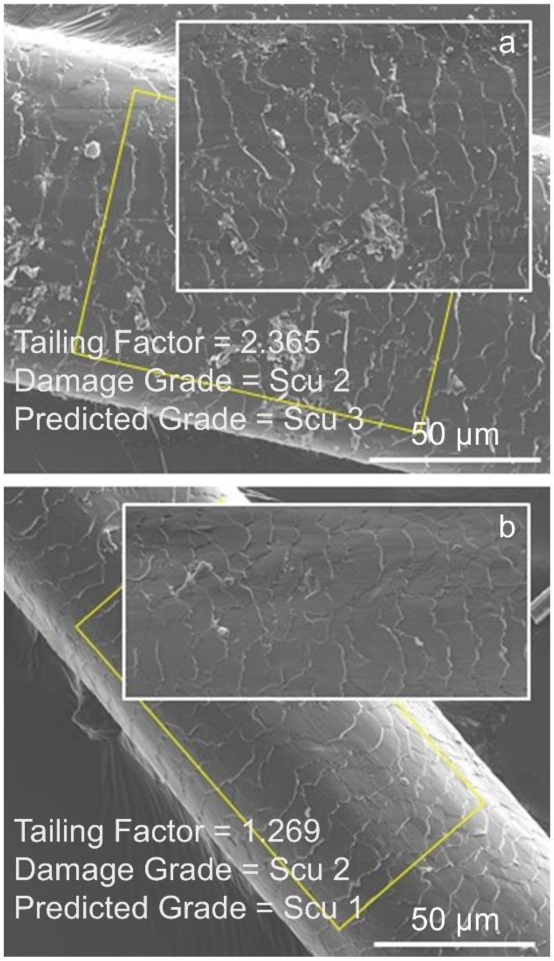

Figure S2. Example SEM images of hair segments with normalized regions of interest from (a) Hair Sample 1 (exploded) and (b) Hair Sample 5 (control) whose damage grades were incorrectly predicted by the kNN model.

Appendix 1. ImageJ macros (for version 1.52k) to normalize SEM images of hair fibers and quantify hair surface damage using tailing factor.

Hair Damage Quantification Menu Tool:

1. Duplicate and Crop Action Tool – duplicates raw image input (TIFF file) and crops out SEM image acquisition parameter label
2. Measure Angle and Rotate Action Tool – measures angle of hair segment with respect to transverse axis and rotates image so that hair segment lies along the transverse axis
3. Normalize Image Action Tool – defines region of interest (ROI) according to Eqs. 2.1 – 2.3 and normalizes ROI according to Eqs. 2.4 and 2.5
4. Calculate Histogram Peak Tailing Action Tool – calculates peak lag tailing from brightness histogram of normalized ROI at 2% of peak height maximum according to Eqs. 2.10 – 2.12

```
//series of macros containing duplicate and crop (to remove SEM image label), measure angle and rotate (to orient
//hair length to lie along the transverse axis), normalize image, and calculate peak tailing factor (to quantify hair
//surface damage)
var sCmds = newMenu("Hair Damage Quantification Menu Tool", newArray("Duplicate and Crop Action Tool",
"Measure Angle and Rotate Action Tool", "-", "Normalize Image Action Tool", "Calculate Histogram Peak Tailing
Action Tool"));

//menu macro to run selected macro

macro "Hair Damage Quantification Menu Tool - Cf00T1a12HC2a0T4c12DC000T7g12Q" {
	cmd = getArgument();
	if (cmd!="-")
		run(cmd);
}

//duplicate and crop macro
//requires raw image input to be TIFF file
//duplicates SEM image and crops label after identifying where label begins (a horizontal line composed entirely of
//white pixels at 255)

macro "Duplicate and Crop Action Tool" {
	orig_name = getTitle();
	//duplicates image
	run("Duplicate...", "  ");
	new_name = replace(orig_name, ".tif", "_ImageJ.tif");
	//renames duplicated image
	rename(new_name);
	//gets dimensions of image in pixels
	image_length = getWidth(); //hair segment length along transverse axis
	image_height = getHeight(); //hair segment width along vertical axis
	//scans image line by line horizontally to find start of label
	for (j=0; j<image_height; j++) {
		makeLine(0, j, image_length, j);
		profile = getProfile();
		max_value = 0;
		min_value = 255;
		for (i=0; i<profile.length; i++) {
```

```
                if (max_value<profile[i]) {
                    max_value = profile[i];
                }
                if (min_value>profile[i]) {
                    min_value = profile[i];
                }
            }
            //stops scanning when finds start of label
            if (max_value==255 && min_value==255) {
                break;
            }
        }
        //crops image to exclude label
        makeRectangle(0, 0, image_length, j);
        run("Crop");
        selectWindow(new_name);
}

var param_table = "Image Parameters";

//measure angle and rotate macro; adapted from “Measure_Angle_And_Length.txt” macro
//requires line input from user - use Line Tool to draw line segment along edge of hair shaft
//measures angle of user-defined line segment with transverse axis and rotates image so hair shaft length lies along
//transverse axis

macro "Measure Angle and Rotate Action Tool" {
        Roi.remove;
        //pops up dialog box to direct user
        waitForUser("Draw line along edge of hair shaft. Click ok when done");
        //extracts line coordinates
        getLine(x1, y1, x2, y2, lineWidth);
        //if Line Tool not selected or no line input, exits out of macro
        if (selectionType!=5 || x1==-1)
            exit("Straight line selection along edge of hair shaft required");
        getPixelSize(unit, length, height, depth);
        x1*=length; y1*=height; x2*=length; y2*=height;
        //finds angle of line with transverse axis using formula below
        angle = getAngle(x1, y1, x2, y2);
        length = sqrt((x2-x1)*(x2-x1)+(y2-y1)*(y2-y1));
        //creates image parameters table
        Table.create("Image Parameters");
        param_table = Table.title;
        //outputs angle and line length measurements
        Table.set("Parameter", Table.size, "Image Name", param_table);
        Table.set("Parameter Value", Table.size-1, getTitle(), param_table);
        Table.set("Parameter", Table.size, "Angle", param_table);
        Table.set("Parameter Value", Table.size-1, angle, param_table);
        Table.set("Parameter", Table.size, "Line Length", param_table);
        Table.set("Parameter Value", Table.size-1, length, param_table);
        dx = x2-x1;
        dy = y1-y2;
        //calculates angle of rotation
```

```
	if (dx<0)
		angle_rot=angle-180.0;
	else if (dx>=0 && dy>=0)
		angle_rot=angle;
	else
		angle_rot=angle-360.0;
	Table.set("Parameter", Table.size, "Angle of Rotation", param_table);
	Table.set("Parameter Value", Table.size-1, angle_rot, param_table);
	//rotates image based on angle of rotation
	run("Rotate...", "angle=angle_rot grid=1 interpolation=Bilinear stack");
	Table.update;
}

//formula to calculate angle (from “Measure_Angle_And_Length.txt” macro)
function getAngle(x1, y1, x2, y2) {
	q1=0; q2orq3=2; q4=3; //quadrant
	dx = x2-x1;
	dy = y1-y2;
	if (dx!=0)
		angle = atan(dy/dx);
	else {
		if (dy>=0)
		angle = PI/2;
		else
		angle = -PI/2;
	}
	angle = (180/PI)*angle;
	if (dx>=0 && dy>=0)
		quadrant = q1;
	else if (dx<0)
		quadrant = q2orq3;
	else
		quadrant = q4;
	if (quadrant==q2orq3)
		angle = angle+180.0;
	else if (quadrant==q4)
		angle = angle+360.0;
	return angle;
}

//normalize image macro
//requires line input from user - use Line Tool to draw diagonal line spanning two corners of the hair segment
//defines region of interest, centered using 75% of length and width of hair segment as defined by diagonal line (Eqs.
//2.1 – 2.3)
//normalizes each pixel to average brightness along hair length and rescales average brightness to 109 (Eq. 2.4)
//rescales normalized pixel to be bounded between 109 and 255 if normalized value is greater than 109 (Eq. 2.5)

var param_table = Table.title;

macro "Normalize Image Action Tool" {
	Roi.remove;
	updateDisplay();
```

```
	//pops up dialog box to direct user
	waitForUser("Draw diagonal line from edge of one end of hair shaft to edge of other end. Click ok when done");
	//extracts line coordinates
	getLine(x1, y1, x2, y2, lineWidth);
	//exits out of macro if Line Tool not selected or no line input
	if(selectionType!=5 || x1==-1)
		exit("Straight line selection along hair shaft diagonal required");
	image_id = getImageID();
	//extracts length and width of hair segment
	image_length = abs(x2-x1);
	image_height = abs(y2-y1);
	//finds center of image
	image_xcenter =(x1+x2)/2;
	image_ycenter = (y1+y2)/2;
	if (x1<x2) {
		x_min = x1;
		x_max = x2;
	} else {
		x_min = x2;
		x_max = x1;
	}
	if (y1<y2) {
		y_min = y1;
		y_max = y2;
	} else {
		y_min = y2;
		y_max = y1;
	}
	//outputs hair segment parameters
	selectWindow(param_table);
	Table.set("Parameter", Table.size, "Image Center_x", param_table);
	Table.set("Parameter Value", Table.size-1, image_xcenter, param_table);
	Table.set("Parameter", Table.size, "Image Center_y", param_table);
	Table.set("Parameter Value", Table.size-1, image_ycenter, param_table);
	Table.set("Parameter", Table.size, "Segment Length", param_table);
	Table.set("Parameter Value", Table.size-1, image_length, param_table);
	Table.set("Parameter", Table.size, "Segment Height", param_table);
	Table.set("Parameter Value", Table.size-1, image_height, param_table);
	//defines upper left-hand corner, length, and width of ROI (Eqs. 2.1 – 2.3)
	rect_x = round(((x_min)/2)+((x_min)/4)+(x_min+x_max)/8);
	rect_y = round(((y_min)/2)+((y_min)/4)+(y_max+y_min)/8);
	rect_length = round((((x_max)/2)+((x_max)/4)+(x_min+x_max)/8))-rect_x;
	rect_height = round((((y_max)/2)+((y_max)/4)+(y_max+y_min)/8))-rect_y;
	Table.set("Parameter", Table.size, "ROI Length", param_table);
	Table.set("Parameter Value", Table.size-1, rect_length, param_table);
	Table.set("Parameter", Table.size, "ROI Height", param_table);
	Table.set("Parameter Value", Table.size-1, rect_height, param_table);
	Table.set("Parameter", Table.size, "ROI Upper LH_x", param_table);
	Table.set("Parameter Value", Table.size-1, rect_x, param_table);
	Table.set("Parameter", Table.size, "ROI Upper LH_y", param_table);
	Table.set("Parameter Value", Table.size-1, rect_y, param_table);
```

```
//scans through raw ROI to obtain average brightness per line
j = rect_y;
m = rect_x;
k = 0;
profile_avg = newArray(rect_height+1);
while (j<=(rect_y+rect_height)) {
	makeLine(rect_x, j, rect_x+rect_length, j);
	profile = getProfile();
	profile_sum = 0;
	m = rect_x;
	while (m<(rect_x+rect_length)) {
		m=m+1;
		profile_sum += profile[m-rect_x];
	}
	profile_avg[k] = profile_sum/profile.length;
	profile_sum_ROI += profile_sum;
	j=j+1;
	k=k+1;
}
//scans through raw ROI to obtain maximum brightness value after normalizing to average line brightness
//and rescaling average to 109 (Eq. 2.4)
j = rect_y;
k = 0; //row_index
i = 0; //col_index
max_value = 0;
profile_normval = newArray(rect_length+1);
while (j<=(rect_y+rect_height)) {
	profile_normval = newArray(rect_length+1);
	makeLine(rect_x, j, rect_x+rect_length, j);
	profile = getProfile();
	m = rect_x;
	i = 0;
	while (m<(rect_x+rect_length)) {
		profile_normval[i] = (profile[i]/profile_avg[k])*109;
		if (profile_normval[i]>max_value) {
			max_value=profile_normval[i];
		}
		m=m+1;
		i=i+1;
	}
	j=j+1;
	k=k+1;
}
//scans through raw ROI to apply normalization and rescaling to raw values (Eq. 2.4 and 2.5)
run("Clear Results");
j = rect_y;
k = 0; //row_index
i = 0; //col_index
profile_newval = newArray(rect_length+1);
profile_newval_avg = newArray(rect_height+1);
while (j<=(rect_y+rect_height)) {
	profile_newval = newArray(rect_length+1);
```

```
			makeLine(rect_x, j, rect_x+rect_length, j);
			profile = getProfile();
			m = rect_x;
			i = 0;
			profile_newval_sum = 0;
			while (m<(rect_x+rect_length)) {
				if ((profile[i]/profile_avg[k])*109>109) {
					profile_newval[i] = 109+(146*(((profile[i]/profile_avg[k])*109)-109)/(max_value-
109));
				} else {
					profile_newval[i] = (profile[i]/profile_avg[k])*109;
				}
				//generates array of normalized brightness values in Results Table (separate from
				//param_table)
				setResult(m, k, profile_newval[i]);
				profile_newval_sum += profile_newval[i];
				m=m+1;
				i=i+1;
			}
			profile_newval_avg[k] = profile_newval_sum/profile.length;
			profile_newval_sum_ROI += profile_newval_sum;
			j=j+1;
			k=k+1;
		}
		updateResults();
		//sets brightness bounds between 0 and 255 in normalized ROI by temporarily converting 2 pixels to 0 and
		//255, respectively
		black_pix_x = rect_x;
		white_pix_x = rect_x+1;
		black_pix_orig = getResult(black_pix_x, 0);
		white_pix_orig = getResult(white_pix_x, 0);
		setResult(black_pix_x, 0, 0);
		setResult(white_pix_x, 0, 255);
		updateResults();
		selectWindow("Results");
		//converts array of normalized brightness values to 32-bit grayscale image
		run("Results to Image");
		//resets 2 pixels to normalized brightness values
		setPixel(black_pix_x-rect_x, 0, black_pix_orig);
		setPixel(white_pix_x-rect_x, 0, white_pix_orig);
		updateDisplay();
		setResult(black_pix_x, 0, black_pix_orig);
		setResult(white_pix_x, 0, white_pix_orig);
		updateResults();
		IJ.renameResults("Normalized ROI Array");
		run("Clear Results");
		//calculates raw and normalized ROI average % brightness
		profile_sum_ROI_avg = profile_sum_ROI/(profile.length*profile_avg.length);
		profile_sum_ROI_avg_percent = (profile_sum_ROI_avg/255)*100;
		profile_newval_sum_ROI_avg = profile_newval_sum_ROI/(profile_newval_avg.length*profile.length);
		profile_newval_sum_ROI_avg_percent = (profile_newval_sum_ROI_avg/255)*100;
		//outputs raw and normalized ROI average % brightness
```

```
		selectWindow(param_table);
		Table.set("Parameter", Table.size, "Raw Average % Brightness", param_table);
		Table.set("Parameter Value", Table.size-1, profile_sum_ROI_avg_percent, param_table);
		Table.set("Parameter", Table.size, "Normalized Average % Brightness", param_table);
		Table.set("Parameter Value", Table.size-1, profile_newval_sum_ROI_avg_percent, param_table);
		Table.update;
		close("Normalized ROI Array");
		selectWindow("Results Table");
		rename("Normalized ROI");
}

//calculate histogram peak tailing macro
//defines peak apex to reduce histogram skew in event of multiple peaks (Eqs. 2.10 and 2.11)
//calculates tailing factor at 2% of peak height maximum (Eq. 2.12)

macro "Calculate Histogram Peak Tailing Action Tool" {
		selectWindow("Normalized ROI");
		//generates histogram of normalized ROI to find peak height maximum H and average brightness value
		q = 0;
		nBins = 256;
		peak_height_max = 0;
		average_brightness_sum = 0;
		pixel_count = 0;
		getHistogram(values, counts, nBins, 0, 255);
		for (q=0; q<nBins; q++) {
				if (counts[q]>peak_height_max) {
						peak_height_max=counts[q];
						brightness_max=q;
				}
				average_brightness_sum+=(counts[q]*q);
				pixel_count+=counts[q];
		}
		average_brightness = average_brightness_sum/pixel_count;
		//calculates peak lead and lag brightness values at full width half maximum for application to Eq. 2.10
		//in event that brightness values are not integers, exact values are calculated using equation of a line from
		//consecutive brightness values that bound the height at half maximum
		height_halfmax = 0.5*peak_height_max;
		for (r=255; r>brightness_max; r--) {
				if ((counts[r]<=height_halfmax) && (counts[r-1]>=height_halfmax)) {
						halfmax_lag_brightness = r;
						halfmax_lag_brightness2 = r-1;
						break;
				}
		}
		slope = (counts[halfmax_lag_brightness]-counts[halfmax_lag_brightness2])/(1);
		intercept = counts[halfmax_lag_brightness]-(slope*(halfmax_lag_brightness));
		halfmax_lag_brightness_exact = (height_halfmax-intercept)/slope;
		for (r=0; r<brightness_max; r++) {
				if ((counts[r]<=height_halfmax) && (counts[r+1]>=height_halfmax)) {
						halfmax_lead_brightness = r;
						halfmax_lead_brightness2 = r+1;
						break;
```

```
		}
	}
	slope = (counts[halfmax_lead_brightness2]-counts[halfmax_lead_brightness])/(1);
	intercept = counts[halfmax_lead_brightness2]-(slope*(halfmax_lead_brightness2));
	halfmax_lead_brightness_exact = (height_halfmax-intercept)/slope;
	//calculates averge brightness value between peak lead and lag at full width half maximum (Eq. 2.10)
	k = round(halfmax_lead_brightness_exact);
	average_brightness_halfmax_sum = 0;
	pixel_count_halfmax_width = 0;
	while (k<=round(halfmax_lag_brightness_exact)) {
		average_brightness_halfmax_sum+=(counts[k]*k);
		pixel_count_halfmax_width+=counts[k];
		k=k+1;
	}
	average_brightness_halfmax = average_brightness_halfmax_sum/pixel_count_halfmax_width;
	//redefines peak apex by comparing brightness at peak height maximum to average brightness when
	//calculated between full width half maximum (Eq. 2.11)
	selectWindow(param_table);
	if (brightness_max<=(average_brightness_halfmax+3) && brightness_max>=(average_brightness_halfmax-
3)) {
		Table.set("Parameter", Table.size, "Brightness_Peak Height Maximum", param_table);
		Table.set("Parameter Value", Table.size-1, brightness_max, param_table);
		Table.set("Parameter", Table.size, "Brightness_Peak Apex", param_table);
		Table.set("Parameter Value", Table.size-1, brightness_max, param_table);
	} else {
		Table.set("Parameter", Table.size, "Brightness_Peak Height Maximum", param_table);
		Table.set("Parameter Value", Table.size-1, brightness_max, param_table);
		brightness_max = round(average_brightness_halfmax);
		Table.set("Parameter", Table.size, "Brightness_Peak Apex", param_table);
		Table.set("Parameter Value", Table.size-1, brightness_max, param_table);
	}
	//calculates peak lead and lag brightness values at 0.02H for application to Eq. 2.12
	//in event that brightness values are not integers, exact values are calculated using equation of a line from
	//consecutive brightness values that bound height at 0.02H
	height_tailing = 0.02*peak_height_max;
	for (r=255; r>brightness_max; r--) {
		if ((counts[r]<=height_tailing) && (counts[r-1]>=height_tailing)) {
			tailing_lag_brightness = r;
			tailing_lag_brightness2 = r-1;
			break;
		}
	}
	slope = (counts[tailing_lag_brightness]-counts[tailing_lag_brightness2])/(1);
	intercept = counts[tailing_lag_brightness]-(slope*(tailing_lag_brightness));
	tailing_lag_brightness_exact = (height_tailing-intercept)/slope;
	for (r=0; r<brightness_max; r++) {
		if ((counts[r]<=height_tailing) && (counts[r+1]>=height_tailing)) {
			tailing_lead_brightness = r;
			tailing_lead_brightness2 = r+1;
			break;
		}
	}
```

```
	slope = (counts[tailing_lead_brightness2]-counts[tailing_lead_brightness])/(1);
	intercept = counts[tailing_lead_brightness2]-(slope*(tailing_lead_brightness2));
	tailing_lead_brightness_exact = (height_tailing-intercept)/slope;
	//calculates tailing factor at 0.02H (Eq. 2.12)
	tailing_factor = (tailing_lag_brightness-tailing_lead_brightness)/(2*(brightness_max-
tailing_lead_brightness));
	//outputs peak lag tailing parameters
	selectWindow(param_table);
	Table.set("Parameter", Table.size, "Peak Height Maximum", param_table);
	Table.set("Parameter Value", Table.size-1, peak_height_max, param_table);
	Table.set("Parameter", Table.size, "Tailing_Peak Lead Brightness", param_table);
	Table.set("Parameter Value", Table.size-1, tailing_lead_brightness_exact, param_table);
	Table.set("Parameter", Table.size, "Tailing_Peak Lag Brightness", param_table);
	Table.set("Parameter Value", Table.size-1, tailing_lag_brightness_exact, param_table);
	Table.set("Parameter", Table.size, "Tailing Factor_0.02H", param_table);
	Table.set("Parameter Value", Table.size-1, tailing_factor, param_table);
	Table.update;
}
```

Appendix 2. ImageJ code (for version 1.52k) to quantify hair surface damage using image roughness (in lieu of macro 4 in Appendix 1).

```
//finds average roughness of image where roughness across the normalized ROI is binned into pixel-by-pixel, 10, 20,
//50, or 100 sections
//performs calculations after normalizing ROI (in lieu of calculating tailing factor)
var param_table = "Image Parameters";
selectWindow("Normalized ROI");
rect_length = getWidth();
rect_height = getHeight()-1;
//determines number of pixels binned to each section (section width) according to Eq. 2.7
sect10_length = floor(rect_length/10)+1;
sect20_length = floor(rect_length/20)+1;
sect50_length = floor(rect_length/50)+1;
sect100_length = floor(rect_length/100)+1;
j = 0; //height index
k = 0; //row_index
p = 0; //col_index
roughness_pixpix_array = newArray(rect_height+1);
roughness_pixpix_sum = 0;
roughness_sect100_array = newArray(rect_height+1);
roughness_sect100_sum = 0;
roughness_sect50_array = newArray(rect_height+1);
roughness_sect50_sum = 0;
roughness_sect20_array = newArray(rect_height+1);
roughness_sect20_sum = 0;
roughness_sect10_array = newArray(rect_height+1);
roughness_sect10_sum = 0;
//calculates roughness across each row of the image according to Eq. 2.9
while (j<=(rect_height)) {
		makeLine(0, j, rect_length, j);
		profile = getProfile();
		m = 0; //length index
		p = 0;
		r = 0; //index for distance_pixpix_array
		u = 1; //index for distance_sect100_array
		v = 1; //index for distance_sect50_array
		w = 1; //index for distance_sect20_array
		z = 1; //index for distance_sect10_array
		distance_pixpix_sum = 0;
		distance_pixpix_array = newArray(rect_length);
		distance_sect10_sum = 0;
		distance_sect10_array = newArray((floor(rect_length/sect10_length)+1));
		distance_sect20_sum = 0;
		distance_sect20_array = newArray((floor(rect_length/sect20_length)+1));
		distance_sect50_sum = 0;
		distance_sect50_array = newArray((floor(rect_length/sect50_length)+1));
		distance_sect100_sum = 0;
		distance_sect100_array = newArray((floor(rect_length/sect100_length)+1));
		while (m<(rect_length)) {
				if (p>0) {
```

```
				distance_pixpix_array[r] = sqrt(((profile[p]-profile[p-1])*(profile[p]-profile[p-1]))+1);
				distance_pixpix_sum += distance_pixpix_array[r];
				r=r+1;
			}
			if (p == (u*sect100_length) || m == (rect_length - 1)) {
				pix_distance_sq_sect100 = (p-((u-1)*sect100_length))*(p-((u-1)*sect100_length));
				distance_sect100_array[u-1] = sqrt(((profile[p]-profile[(u-1)*sect100_length])*(profile[p]-
profile[(u-1)*sect100_length]))+pix_distance_sq_sect100);
				distance_sect100_sum += distance_sect100_array[(u-1)];
				u=u+1;
			}
			if (p == (v*sect50_length) || m == (rect_length - 1)) {
				pix_distance_sq_sect50 = (p-((v-1)*sect50_length))*(p-((v-1)*sect50_length));
				distance_sect50_array[v-1] = sqrt(((profile[p]-profile[(v-1)*sect50_length])*(profile[p]-
profile[(v-1)*sect50_length]))+pix_distance_sq_sect50);
				distance_sect50_sum += distance_sect50_array[v-1];
				v=v+1;
			}
			if (p == (w*sect20_length) || m == (rect_length - 1)) {
				pix_distance_sq_sect20 = (p-((w-1)*sect20_length))*(p-((w-1)*sect20_length));
				distance_sect20_array[w-1] = sqrt(((profile[p]-profile[(w-1)*sect20_length])*(profile[p]-
profile[(w-1)*sect20_length]))+pix_distance_sq_sect20);
				distance_sect20_sum += distance_sect20_array[w-1];
				w=w+1;
			}
			if (p == (z*sect10_length) || m == (rect_length - 1)) {
				pix_distance_sq_sect10 = (p-((z-1)*sect10_length))*(p-((z-1)*sect10_length));
				distance_sect10_array[z-1] = sqrt(((profile[p]-profile[(z-1)*sect10_length])*(profile[p]-
profile[(z-1)*sect10_length]))+pix_distance_sq_sect10);
				distance_sect10_sum += distance_sect10_array[z-1];
				z=z+1;
			}
			m=m+1;
			p=p+1;
		}
		roughness_pixpix_array[k] = distance_pixpix_sum/distance_pixpix_array.length;
		roughness_pixpix_sum += roughness_pixpix_array[k];
		roughness_sect100_array[k] = distance_sect100_sum/rect_length;
		roughness_sect100_sum += roughness_sect100_array[k];
		roughness_sect50_array[k] = distance_sect50_sum/rect_length;
		roughness_sect50_sum += roughness_sect50_array[k];
		roughness_sect20_array[k] = distance_sect20_sum/rect_length;
		roughness_sect20_sum += roughness_sect20_array[k];
		roughness_sect10_array[k] = distance_sect10_sum/rect_length;
		roughness_sect10_sum += roughness_sect10_array[k];
		j=j+1;
		k=k+1;
}
//calculates average image roughness in ROI
roughness_pixpix_avg = roughness_pixpix_sum/roughness_pixpix_array.length;
roughness_sect100_avg = roughness_sect100_sum/roughness_sect100_array.length;
roughness_sect50_avg = roughness_sect50_sum/roughness_sect50_array.length;
roughness_sect20_avg = roughness_sect20_sum/roughness_sect20_array.length;
```

```
roughness_sect10_avg = roughness_sect10_sum/roughness_sect10_array.length;
selectWindow(param_table);
Table.set("Parameter", Table.size, "Average Roughness Pixel-by-Pixel", param_table);
Table.set("Parameter Value", Table.size-1, roughness_pixpix_avg, param_table);
Table.set("Parameter", Table.size, "Average Roughness 10 Sections", param_table);
Table.set("Parameter Value", Table.size-1, roughness_sect10_avg, param_table);
Table.set("Parameter", Table.size, "Average Roughness 20 Sections", param_table);
Table.set("Parameter Value", Table.size-1, roughness_sect20_avg, param_table);
Table.set("Parameter", Table.size, "Average Roughness 50 Sections", param_table);
Table.set("Parameter Value", Table.size-1, roughness_sect50_avg, param_table);
Table.set("Parameter", Table.size, "Average Roughness 100 Sections", param_table);
Table.set("Parameter Value", Table.size-1, roughness_sect100_avg, param_table);
Table.update;
```
